# Facile Fabrication of Superhydrophobic Graphene/Polystyrene Foams for Efficient and Continuous Separation of Immiscible and Emulsified Oil/Water Mixtures

**DOI:** 10.3390/polym14112289

**Published:** 2022-06-05

**Authors:** Chunxia Zhao, Haoran Huang, Jiaxin Li, Yuntao Li, Dong Xiang, Yuanpeng Wu, Ge Wang, Mingwang Qin

**Affiliations:** 1 School of New Energy and Materials, Southwest Petroleum University, Chengdu 610500, China; HuangHaoran0805@163.com (H.H.); ljx991022@sina.com (J.L.); dxiang01@hotmail.com (D.X.); ypwu@swpu.edu.cn (Y.W.); wangge12335@126.com (G.W.); 2 The Center of Functional Materials for Working Fluids of Oil and Gas Field, Sichuan Engineering Technology Research Center of Basalt Fiber Composites Development and Application, Southwest Petroleum University, Chengdu 610500, China; 3 State Key Laboratory of Oil and Gas Reservoir Geology and Exploitation, Southwest Petroleum University, Chengdu 610500, China; 4 School of Engineering, Southwest Petroleum University, Nanchong 637001, China; QinMingwang123@126.com

**Keywords:** graphene, polystyrene, superhydrophobic, porous materials, oily wastewater treatment

## Abstract

Three-dimensional superhydrophobic/superlipophilic porous materials have attracted widespread attention for use in the separation of oil/water mixtures. However, a simple strategy to prepare superhydrophobic porous materials capable of efficient and continuous separation of immiscible and emulsified oil/water mixtures has not yet been realized. Herein, a superhydrophobic graphene/polystyrene composite material with a micro-nanopore structure was prepared by a single-step reaction through high internal phase emulsion polymerization. Graphene was introduced into the polystyrene-based porous materials to not only enhance the flexibility of the matrix, but also increase the overall hydrophobicity of the composite materials. The resulting as-prepared monoliths had excellent mechanical properties, were superhydrophobic/superoleophilic (water/oil contact angles were 151° and 0°, respectively), and could be used to continuously separate immiscible oil/water mixtures with a separation efficiency that exceeded 99.6%. Due to the size-dependent filtration and the tortuous and lengthy micro-nano permeation paths, our foams were also able to separate surfactant-stabilized water-in-oil microemulsions. This work demonstrates a facile strategy for preparing superhydrophobic foams for the efficient and continuous separation of immiscible and emulsified oil/water mixtures, and the resulting materials have highly promising application potentials in large-scale oily wastewater treatment.

## 1. Introduction

Treatment of oily wastewater has attracted growing attention from the academic and industrial sectors in recent years due to the growing global environmental hazards associated with the discharge of industrial and domestic sewage as well as oil spills [1,2]. Traditional disposal techniques used to treat oily wastewater, such as gravity-driven separation, oil skimmers, in situ burning, methods that rely on centrifugal separation, dispersion with chemical reagents and microbial treatments, have low efficiencies, high costs and can even cause secondary pollution to the environment [3,4,5]. Moreover, treatment of oily wastewater composed of surfactant-stabilized emulsions is even more challenging [6,7,8,9,10]. Therefore, novel techniques and materials that efficiently separate immiscible and emulsified oil/water mixtures are needed for practical use in wastewater treatment.

The high porosity, interconnected pore structure and unique wettability of porous materials make them highly valued in oily wastewater treatment [11,12]. Moreover, the surface wettability of porous materials can be adjusted to tune their selectivity and separation ability for oil/water mixtures [13,14,15,16,17,18,19,20]. For example, Li et al. [21] modified Fe_3_O_4_ nanoparticles with siloxanes and then used the modified nanoparticles to adjust the hydrophobicity of polyurethane foams by a simple drop-coating method. The resulting magnetic materials were able to remotely magnetically absorb oil from water and achieve gravity-driven oil/water separation. Kang et al. [22] prepared a superhydrophobic/superoleophilic material based on the surface modification of wood fibers that showed excellent performance in oil/water separations. Moreover, the prepared material could be used in multiple environments and achieved highly efficient absorption- and filtration-based separation of oil/water mixtures. While these materials were able to absorb oil from wastewater, treating large-scale oil spills with such types of oil sorbents is a time-consuming and labor-intensive task. A large amount of absorbent material is often required because the absorbents have a limited capacity for the oils [23]. Moreover, absorbent-based separations are not scalable for large-scale oily wastewater treatment because the oil/water mixtures must be collected before it can be filtered [24,25,26,27]. Therefore, in order to simplify the separation process, it is necessary to design and manufacture devices based on oil absorbing materials that can continuously and efficiently handle oily wastewater. Towards this goal, Ge et al. developed an in situ oil collection device that was aided by the application of external force [28]. Such continuous oil collection processes require oil absorbing materials with high mechanical performances as well as excellent hydrophobic and lipophilic properties [29]. Many strategies have been developed for the modification of commercial melamine foams, polyurethane foams or metal foams (pore sizes ranging from 100 to 500 µm) to create materials with the desired characteristics for the continuous clean-up of large-scale oil spills [22,30,31,32]. Although the design of these superwetting foams is exquisite, the pore structure of the matrix material is not sufficient to act as particle sieves and separate emulsions, especially emulsions with particle sizes less than 20 µm [33,34,35]. On the other hand, the surface modification of the matrix material requires either complicated procedures or expensive equipment, and even fluorochemicals are utilized to reduce the surface energy, causing secondary environmental pollution. Therefore, the exploration of new strategies for the preparation of superwettable materials with controlled pore structures is needed to realize the continuous and efficient separation of immiscible and emulsified oil/water mixtures.

In recent years, the high internal phase emulsion (HIPE) templating method has been studied extensively to prepare polymer-based porous materials with adjustable pore structures and porosities [36,37,38,39,40,41,42,43]. However, the HIPE templating method is typically performed using rigid monomers, and the resulting porous materials have poor mechanical properties in that they are highly brittle and easily pulverized [44,45]. In addition, the hydrophobic properties of such materials need to be further improved for practical applications in handling oily wastewater [46]. To address these limitations, here we prepare graphene/polystyrene (GN/PSt) porous materials via the HIPE templating method. The introduction of graphene simultaneously improves the flexibility and enhances the hydrophobic properties of the polymer-based matrix. The as-prepared GN_4_/PSt superhydrophobic porous materials achieved in situ continuous absorption of heavy and light oils from water with separation efficiencies that exceeded 99.6%. Moreover, the micro-nanopore structure and wettability of the prepared materials allowed for the efficient and continuous separation of surfactant-stabilized water-in-oil microemulsions. These advantages demonstrate the potential value of our prepared foams for the large-scale and continuous separation of immiscible and emulsified oil/water mixtures.

## 2. Materials and Methods

### 2.1. Materials

Styrene (St) and Span 80 were obtained from Innochem Technology Co., Ltd., Beijing, China. Divinylbenzene (DVB), sodium persulfate (Na_2_S_2_O_8_) and sodium dodecyl sulfonate (SDS) were obtained from Aldrich, Shanghai, China. Graphene (GN, lamellae size 5–15 µm) was supplied by Deyang Carbonene Technology Co., Ltd., and the lamellae were composed of five to six layers of graphene. Absolute ethanol, petroleum ether, chloroform, toluene and acetone were all purchased from Chengdu Kelong Chemical Reagent Factory. Ultrapure deionized water from an ultrapure water machine was used to prepare all solutions.

### 2.2. Fabrication of GN/PSt Foams

**Figure 1:**
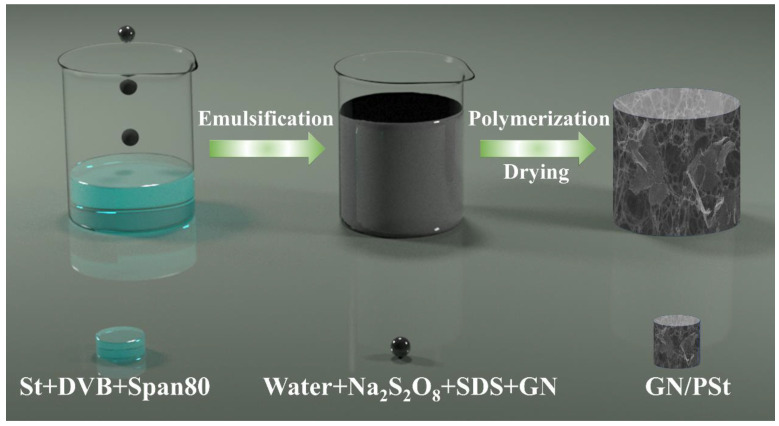
Schematic illustration of the fabrication of superhydrophobic GN/PSt foams.

In a typical preparation process, 0.02 g of Na_2_S_2_O_8_, 0.004 g of SDS and 0.02 g of GN were ultrasonically dispersed in 20 mL deionized water to obtain a homogeneous solution. This solution was then gradually added to a beaker containing 0.3 g of St, 0.2 g of DVB and 0.15 g of Span 80, and the mixture was stirred at 200 rpm until a homogeneous and viscous solution was formed, which was the HIPE pre-polymerization mixture. The beaker was sealed and kept at 65°C for 8 h to allow the polymerization reaction to proceed. After polymerization, the product was washed with absolute ethanol to remove the Span 80 and unreacted organic monomers. The obtained gray monolith was dried at 50 °C for 10 h in a blast oven. The prepared superhydrophobic polystyrene-based porous material containing 4 wt% GN was labeled as GN_4_/PSt. Samples containing 0 wt%, 2 wt%, 8 wt% and 10 wt% GN were prepared following the same protocol and were labelled as GN_0_/PSt, GN_2_/PSt, GN_8_/PSt and GN_10_/PSt, respectively. The fabrication process is schematically illustrated in Figure 1.

### 2.3. Characterization

The microscopic pore structures in the prepared materials were characterized with scanning electron microscopy (SEM, JEOL JSM-5009LV), and the pore size distribution and porosity of the GN_4_/PSt composites were determined using an automatic mercury porosimeter (Mike 9500). The water contact angles (WCA), rolling angles (RA) and oil contact angles (OCA) were characterized using a contact angle goniometer (OCA 25, Data physics Instruments GmbH, Germany). Contact angle measurements were made by dropping 3 μL of deionized water or petroleum ether on three different locations of each sample surface, and the average value is presented. The compressive properties of the porous materials were evaluated using a universal material testing machine (CMT4104, MTS, USA) with a compression rate of 2 mm/min. Samples used in the compressive tests were cylindrical with a height of 20 ± 2 mm and a diameter of 24 mm.

#### 2.3.1. Oil Absorption Capacity

The saturated oil absorption capacity (*k*) of the GN_4_/PSt superhydrophobic porous material was calculated according to Equation (1) [12]:

(1) $$k=\frac{m_{1}-m_{0}}{m_{0}}$$

where *m*_0_ is the original mass of the GN_4_/PSt porous material, and *m*_1_ is the mass of the GN_4_/PSt porous material saturated with oil.

#### 2.3.2. Oil/Water Mixture Separation Efficiency

The model oil/water mixtures were continuously separated using the prepared porous materials with the aid of an external pump, and the separation efficiency (η) was calculated following Equation (2):

(2) $$\eta \;=\;\frac{m_{a}}{m_{b}}\;\times \;100\%\;$$

where *m_b_* and *m_a_* denoted the water mass before and after separation, respectively.

#### 2.3.3. Emulsified Oil/Water Mixture Separation Experiments

Three surfactant-stabilized water-in-oil (W/O) emulsions (water/petroleum ether; water/toluene; water/chloroform) were prepared to simulate stable oil/water mixtures found in real world applications. The emulsions were prepared by mixing water and oil (V_water_: V_oil_ = 1:100) with 1 g/L Span 80 under vigorous stirring for 1 h. The resulting emulsions were stable for at least 24 h.

Continuous oil/water emulsion separation experiments were performed with the aid of an external power source. Microscopic images of the emulsions before and after separation with the GN/PSt composites were taken with an eyepiece inverted fluorescent digital microscope (AMG EVOSFL, USA).

The separation efficiency (*E*) of the oil/water emulsion was calculated according to Equation (3) [47]:

(3) $$E=(1-\frac{C_{s}}{C_{0}})\;\times \;100\%\;$$

where *C_0_* and *Cs* represent the moisture content in the emulsion and the filtrate, respectively, and the moisture contents in the oil were determined using a Karl Fischer moisture meter (Mettler V10S).

## 3. Results and Discussion

### 3.1. Characterizations of the GN/PSt Composites

**Figure 2:**
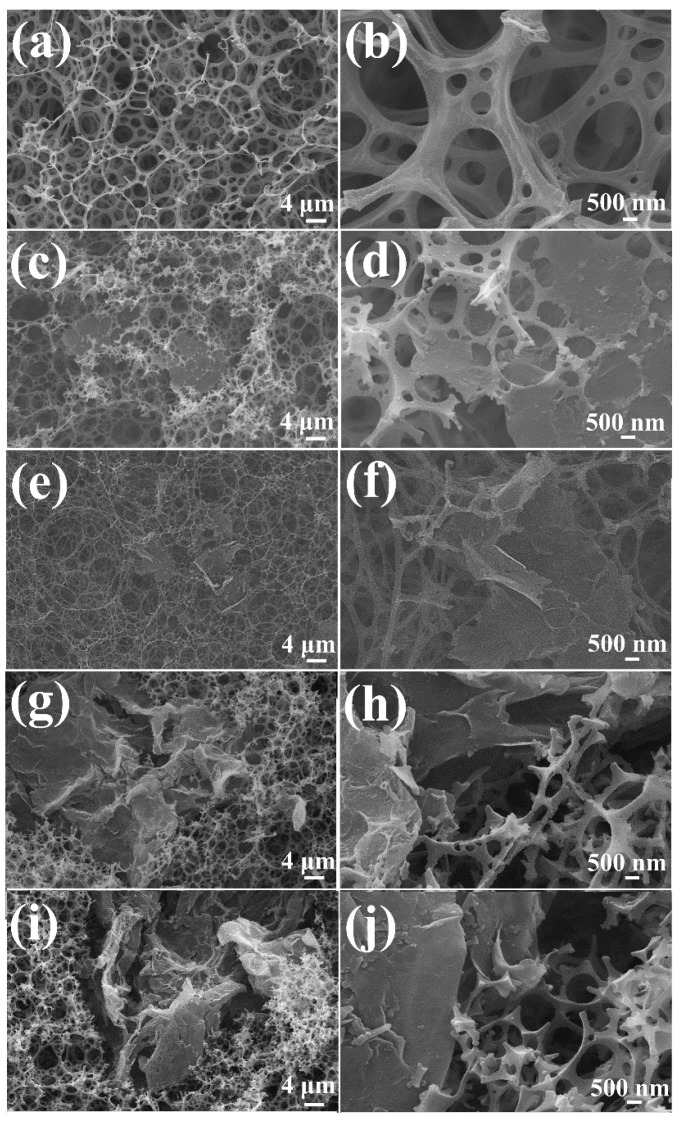
SEM images of the GN_0_/PSt (**a**,**b**), GN_2_/PSt (**c**,**d**), GN_4_/PSt (**e**,**f**), GN_8_/PSt (**g**,**h**) and GN_10_/PSt (**i**,**j**) composites.

During HIPE, water was dispersed in an organic continuous phase consisting of St and DVB, forming a water-in-oil emulsion stabilized by the lipophilic surfactant, Span 80, co-Pickering-surfactant and GN nanosheets. The organic phase, St and DVB, was then polymerized to form the skeleton of the porous material [48], and the water phase was removed from the pore cavities to give the GN/PSt porous materials with high porosities and open pore structures (Figure 2). As seen in the SEM images in Figure 2a, the GN/PSt composites contained interconnected polymer walls that formed large spherical pores (Figure 2a), and many smaller pores were present within the polymer skeleton (Figure 2b) [49]. GN randomly penetrated into the matrix of the porous materials, resulting in a rougher microstructure and lower surface energy (Figure 2c–f) [50]. Similar interconnected porous structures were seen in the GN_0_/PSt, GN_2_/PSt and GN_4_/PSt composites, which should be advantageous for the better transfer of substances through the porous materials when the monoliths are used to separate oil/water mixtures. Meanwhile, a more closed-cell structure was seen in the GN/PSt composites prepared with 8% and 10% GN (Figure 2g–j) because the GN agglomerated and was harder to disperse at these high concentrations and such a closed cell structure is not conducive to the transfer of matter through the materials.

Moreover, the Raman spectra of GN and the GN_4_/PSt composite are shown in Figure S1. Two obvious peaks corresponding to D and G bands appeared at approximately 1358 cm^−1^ and 1586 cm^−1^, respectively. The G band was generally associated with the E_2g_ phonon of the C sp^2^ atom, while the D band arose from the activation of the first-order scattering process of the sp^3^ carbon atom in graphene sheets [51]. In the GN_4_/PSt composite, the same characteristic peaks as GN clearly appeared. This result demonstrated that the composite was successfully prepared. Furthermore, the intensity ratio of D bands to G bands (ID/IG) is usually adopted to evaluate the defects of graphene sheets. The ID/IG of GN used in this experiment was approximately 0.1071, and it increased substantially to 0.1897 for the GN_4_/PSt composite. The higher ID/IG of GN_4_/PSt resulted from a decrease in the crystalline sp^2^ domains of GN, which may be caused by the intercalation of monomer polymerization in the GN layers [52].

### 3.2. Hydrophobicity of the GN/PSt Composites

**Figure 3:**
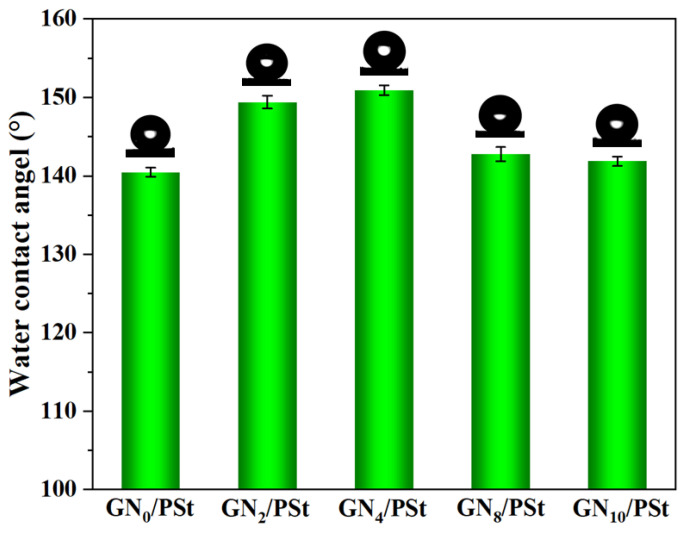
WCAs of the GN/PSt composite porous materials.

The wettability of the GN/PSt porous materials was quantified by their water contact angles. As shown in Figure 3, the WCA of the GN/PSt composite foams was higher than that of the pure foam prepared without GN or GN_0_/PSt (WCA—140.5 ± 0.7°), suggesting that the addition of the GN nanosheets made the PSt-based foams more hydrophobic. The highest WCA was measured for the GN_4_/PSt composite monolith (WCA—150.9 ± 0.6°), indicating that it was the most hydrophobic of the prepared materials.

### 3.3. Mechanical Properties of GN/PSt Composites

**Figure 4:**
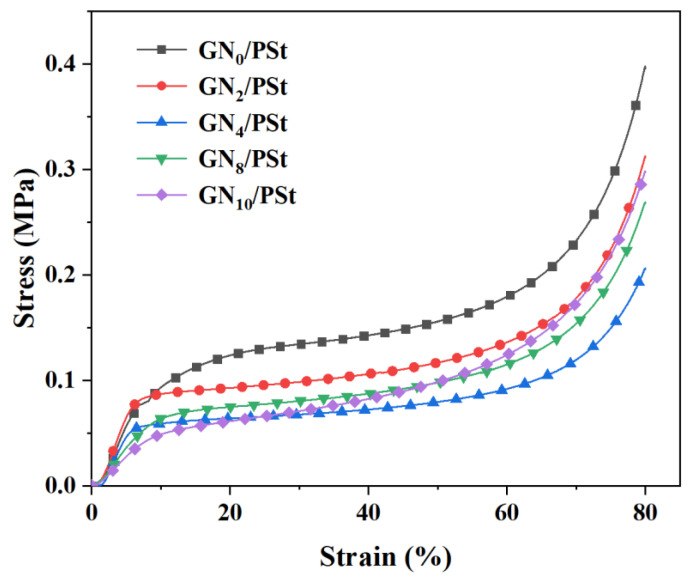
Compressive stress–strain curve measured of the composite porous materials.

To be useful in practical applications, the porous material used for oil/water separation processes must have excellent mechanical properties [53]. Figure 4 shows that the overall trends in the compressive stress–strain curves measured for the GN/PSt composites with different GN contents were similar, and the curves could be divided into three stages. At strains less than 10%, the stress increased linearly with the applied strain. Between 10% and 60% strain, the measured stress plateaued, indicating that the porous material absorbed a large amount of compressive energy and suggesting that the skeleton of the structure was highly deformed. At strains greater than 60%, the material began to fracture under the applied pressure and underwent a densification process. At this time, the stress increased sharply with the increase in the strain [54]. Materials prepared by HIPE polymerization have poor mechanical properties, for example, they are highly brittle and easily pulverized, due to the use of rigid monomers and the high crosslinking densities of the final materials [44]. Here, we see that as the amount of GN added during the HIPE polymerization increased, the compressive strength of the resulting composite materials first decreased and then increased. Small amounts of added GN increased the flexibility of the material, but the addition of excessive amounts of GN resulted in uneven dispersion and agglomeration of the nanosheets, which concentrated the stress and reduced the flexibility of the final composite material. From the present studies, the addition of 4% GN was the optimal amount to effectively improve the flexibility of the porous material while also increasing its hydrophobicity. Therefore, GN4/PSt was selected for the subsequent tests.

### 3.4. Wettability of GN_4_/PSt Composites

**Figure 5:**
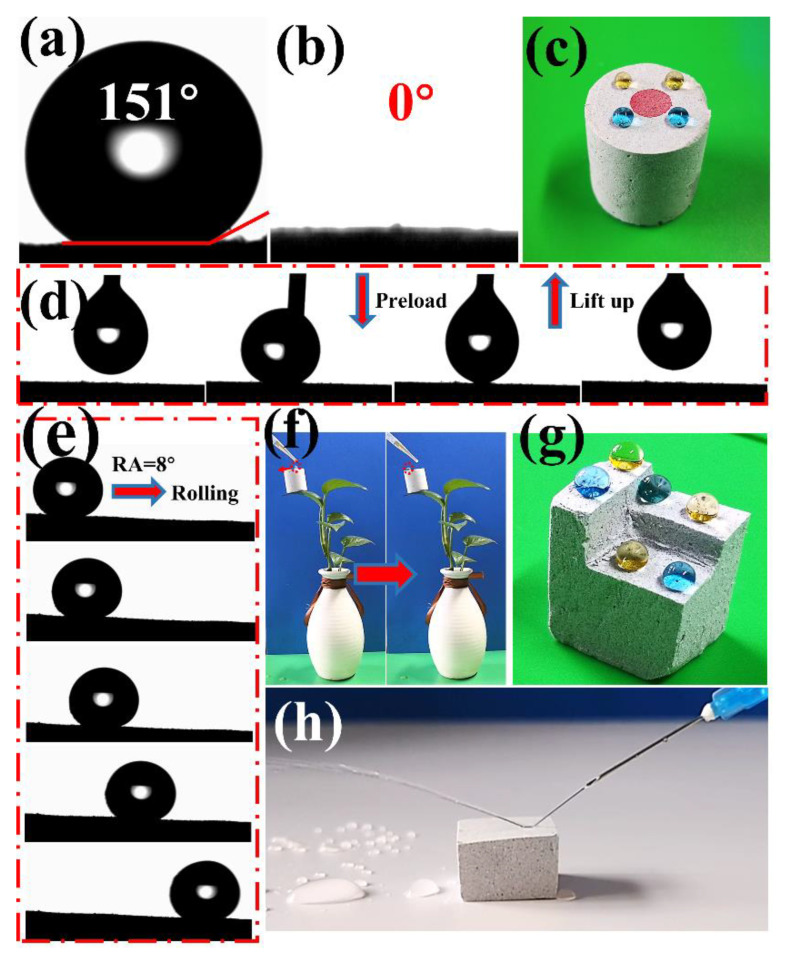
(**a**) WCA and (**b**) OCA of GN_4_/PSt, (**c**) photographs of water (dyed with brilliant green and methyl orange) and oil (dyed with oil red O) droplets on the surface of GN_4_/PSt, (**d**) dynamic adhesion behavior of water droplets on GN_4_/PSt surface, (**e**) the rolling angle, (**f**) water droplets falling on the surface of GN_4_/PSt, (**g**) water droplets on a random surface of GN_4_/PSt, (**h**) water impact resistance of GN_4_/PSt.

The wettability of porous materials towards water and oil is a vital indicator of their performance in oil/water separations [55]. As seen in Figure 5a,b, the WCA and OCA of the GN_4_/PSt porous composite were 151° and 0°, respectively. To more clearly demonstrate the superhydrophobicity and oleophilicity of the prepared composite, water and oil were dropped onto the surface of the GN_4_/PSt monolith, and the water droplets remained as spherical shapes while the oil droplets were rapidly absorbed (Figure 5c). Under the application of an external force, a water droplet was forced to make contact and move on the surface of the material, and the water droplet moved easily and did not remain on the surface of the foam, suggesting that the water adhesion on the GN_4_/PSt composite was extremely low (Figure 5d). Moreover, the rolling angle (RA) of the material was small (RA = 8°, Figure 5e). As shown in Figure 5f, the lightweight GN_4_/PSt porous material could be placed on a thin blade, and a water droplet placed on the surface easily rolled off at only a slight incline due to the low density and low RA of the prepared composite materials. An automatic mercury porosimeter was used to characterize the pore structure of GN_4_/PSt composites, and the density and porosity were 0.0276 g/cm^3^ and 97.2%, respectively. The results indicated that the GN_4_/PSt foam had a hierarchical pore structure containing both nanopores (40–1000 nm) and micropores (1–20 μm) (ESI; Figure S2), suggesting that the prepared material should be able to separate oil/water emulsions through a size sieving effect [56]. Moreover, the properties of the GN_4_/PSt porous sample were highly uniform, and the monolith could be randomly cut into various segments without affecting the shapes of the water droplets on any of the surfaces (Figure 5g). Figure 5h shows that the GN_4_/PSt porous material also had a strong water-impact resistance [57]. In summary, the addition of GN into an HIPE system resulted in GN_4_/PSt composite materials with superhydrophobic/superoleophilic properties as well as a low density and high porosity, making this material especially promising for the purification of oily sewage.

### 3.5. Oil Absorption Capacity and Continuous Oil/Water Separation Using the GN_4_/PSt Composites

**Figure 6:**
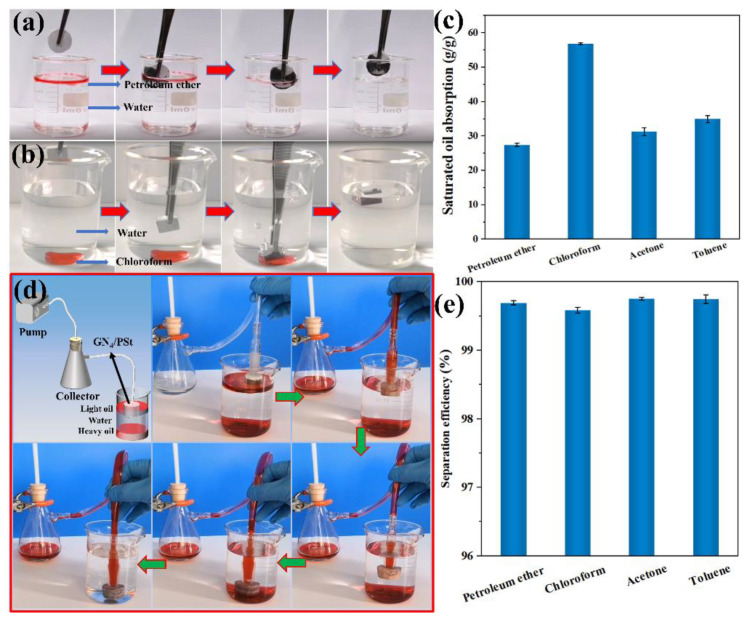
Photographs showing the use of GN_4_/PSt composite materials for selective oil/water separation. (**a**) Petroleum ether/water and (**b**) chloroform/water, (**c**) the saturated oil absorption capacity of GN_4_/PSt foam for a variety of organic solvents, (**d**) images of the continuous separation of light oil/water/heavy oil mixtures using the GN_4_/PSt foams and corresponding (**e**) separation efficiencies for different oil/water mixtures.

As shown in Figure 6a,b, both light (petroleum ether) and heavy (chloroform) oils were rapidly absorbed into the GN_4_/PSt composite by capillary forces, suggesting the prepared composites should show good performance in water/oil separation experiments. The saturated oil absorption capacity of the GN_4_/PSt foams for various types of organic solvents (Figure 6c) and ranged from 27.44 to 56.9 g/g. These results suggested that the prepared composites had a high absorption capacity for various organic solvents, and the variations in the different oil absorption capacities were due to differences in the densities and viscosities of the absorbed organic solvents [58]. The reusability of materials is also a key factor in the actual treatment of oily wastewater. The reusability of GN_4_/PSt foam was examined by absorption-centrifugation. After the oil absorption reached saturation, the oil in the GN_4_/PSt foam was removed by centrifugation at 6000 rpm for 2 min, while the foam was regenerated for the next absorption/centrifugation cycle without further treatment (Figure S3a). The absorption/centrifugation cycle test was repeated 10 times and the results are shown in Figure S3b. The porous material exhibited stable reuse performance with almost no change in oil absorption capacity, and approximately 84% of petroleum ether was removed by each centrifugation. It is speculated that the residual petroleum ether was stably adsorbed and retained in the micropores by van der Waals and capillary forces. In addition, the GN_4_/PSt sample continuously absorb oil pumped through the foam, which is a necessary feature of materials used in large-scale oily wastewater treatment. As shown in Figure 6d and Video S1, both light oil (petroleum ether) and heavy oil (chloroform) were quickly and continuously separated from water without leaving any residual red oil in the water phase. As shown in Figure 6e, the GN_4_/PSt foams maintained a high separation efficiency for various organic solvents in water (above 99.6%), further highlighting that the GN_4_/PSt composite material has great application potential for efficient, large-scale and continuous treatment of oily wastewater.

### 3.6. Continuous Separation of Surfactant-Stabilized Emulsions Using the GN_4_/PSt Composites

**Figure 7:**
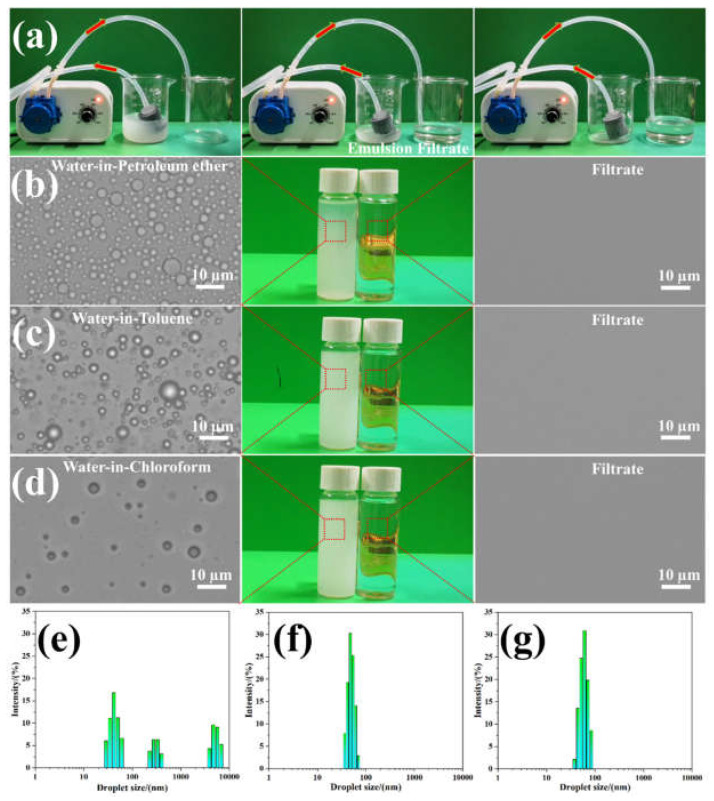
(**a**) Photographs of the setup using GN_4_/PSt composites for the continuous separation of water-in-petroleum ether emulsions and corresponding optical microscopy images and digital photos of the emulsions before and after separation, (**b**) water-in-petroleum ether emulsions, (**c**) water-in-toluene emulsions, (**d**) water-in-chloroform emulsions, (**e**) droplet size distributions in the water-in-petroleum ether emulsion, (**f**) droplet size distribution in the filtrate, and (**g**) droplet size distribution in a solution of 1 g/L Span 80 surfactant in petroleum ether.

The separation of emulsions is much more difficult than the separation of immiscible water/oil mixtures, and the separation of surfactant-stabilized microemulsions with small droplet sizes and lower content of dispersive phase is especially challenging [59]. To test the feasibility of separating oil/water emulsions using the GN_4_/PSt foams, three surfactant-stabilized water-in-oil emulsions with micro-nano particle sizes were prepared, and the separation of Span 80-stabilized water-in-petroleum ether emulsions are discussed as an example. As shown in Figure 7a and Video S2, after the milky white emulsion was pumped through the monolith, the filtrate was clear and transparent indicating that in situ demulsification and continuous oil/water separation were realized. Moreover, optical micrographs and digital photographs of the as-prepared water-in-oil emulsions before and after separation using the GN_4_/PSt foams are shown in Figure 7b–d. While numerous dispersed droplets were seen in the feed solutions, no droplets were observed in the filtrate using an optical microscope, further highlighting the high-efficiency separation performance of the GN_4_/PSt foams. Furthermore, the droplet size of the feed emulsions and filtrates were measured using the dynamic light scattering. The initial emulsions contained a wide size distribution of droplets ranging from 30 nm to 7 µm (Figure 7e). In comparison, the droplets in the filtrate were smaller than 100 nm (Figure 7f), and the particle sizes were similar to those seen in a solution of the Span 80 surfactant in oil (Figure 7g). Based on these results, we speculate that the filtrate was composed of a small number of nano-sized emulsified water droplets and surfactant micelles [56,60].

**Figure 8:**
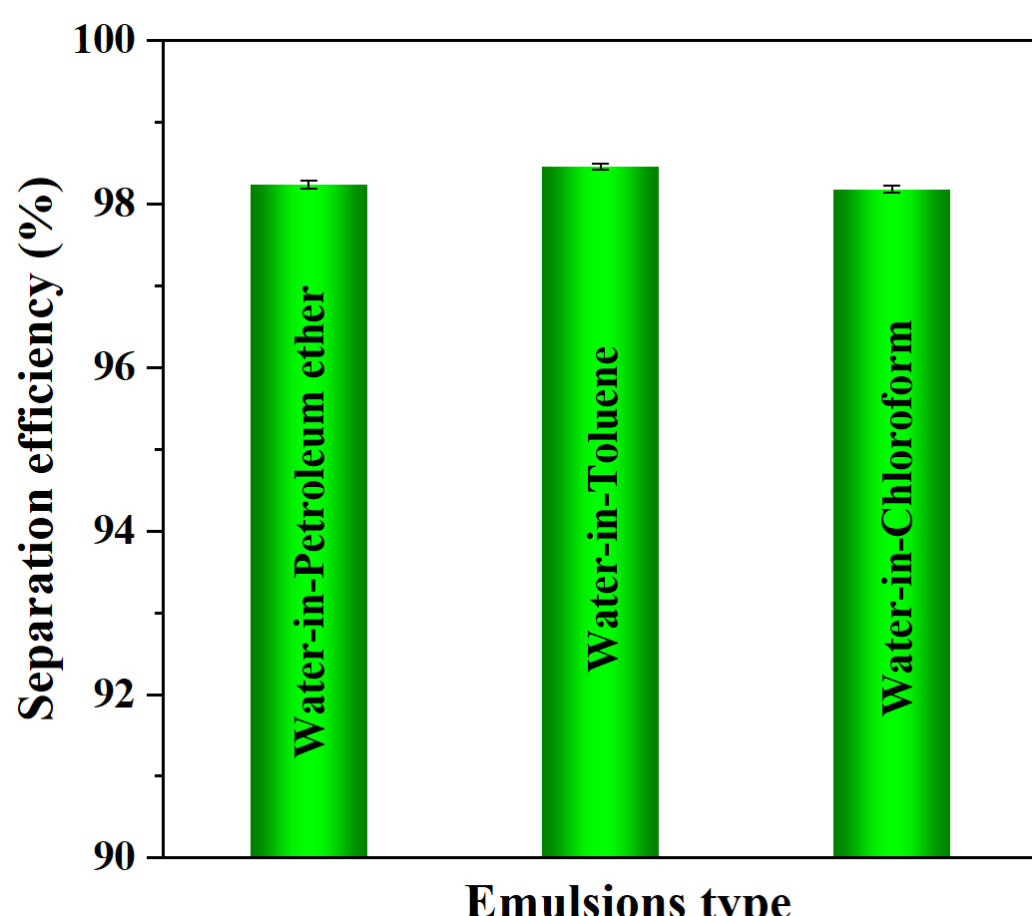
Separation efficiency of water-in-oil emulsions.

To more quantitatively assess the demulsification efficiency of the GN_4_/PSt foams, the moisture contents of the emulsions before and after the separation were measured using a Karl Fischer moisture meter, and the results are shown in Figure 8. The separation efficiencies of the GN_4_/PSt composites for the three emulsions were 98.2%, 98.5% and 98.2%, respectively. This high separation efficiency is due to a combination of size-sieving filtration in the GN_4_/PSt foams as well as the completely opposite wettability towards oil and water. The long and tortuous micro-nano permeation channels in the foams were also crucial in the emulsion separation process [35,61,62]. In summary, the as-prepared superhydrophobic GN_4_/PSt porous materials could be used for the efficient, large-scale and continuous separation of emulsions to achieve rapid treatment of emulsified oily sewage.

## 4. Conclusions

In summary, the superhydrophobic GN_4_/PSt composite material with micro-nanopore structures was successfully fabricated using a facile, low-cost HIPE polymerization method. Compared with a neat porous material prepared with only PSt, the porous monolith prepared with 4 wt% GN was more hydrophobic (WCA increased from 140.5 ± 0.7° to 150.9 ± 0.6°) and more flexible. Due to the ideal wettability, excellent mechanical properties and high porosity (97.2%), the GN_4_/PSt composite monolith could be used to continuously separate immiscible oil/water mixtures with a separation efficiency above 99.6%. Arising from the lengthy micro-nanopore permeation path, the GN_4_/PSt enable efficient and continuous separation of surfactant-stabilized water-in-oil microemulsions. The special wettability, hierarchical pore structure with micro-nanopore and tortuous permeation channel play the significant roles in emulsion separation. The superhydrophobic GN_4_/PSt composite material prepared here showed great performance for the efficient and continuous separation of immiscible and emulsified oil/water mixtures and has promising application prospects in the practical large-scale treatment of oily wastewater.

## Data Availability

Not applicable.

## Supplementary Materials

The following supporting information can be downloaded at: https://www.mdpi.com/article/10.3390/polym14112289/s1, Figure S1. Raman spectra of GN and GN4/PSt.; Figure S2. The pore size distribution of GN4/PSt porous material.; Figure S3. (a) The process of removal of petroleum ether from oil/water mixture and the regeneration of the foam. (b) Regeneration of GN4/PSt sponge during the absorption of petroleum ether for 10 cycles.; Video 1: Continuous separation of immiscible light oil/water/heavy oil by GN4/PSt porous material with the assistance of a pump.; Video 2: Continuous separation of surfactant-stabilized water-in-oil microemulsions with the assistance of a pump.
