# Supplementary material for: Facile Fabrication of Superhydrophobic Graphene/Polystyrene Foams for Efficient and Continuous Separation of Immiscible and Emulsified Oil/Water Mixtures
Source: Polymers (Basel). 2022 Jun 5;14(11):2289. doi: 10.3390/polym14112289 (PMC9183141; doi:10.3390/polym14112289)
Supplement: Supplementary file 1 [file polymers-14-02289-s001.zip › polymers-1720017-supplementary/Revised Supporting information.pdf]

Facile Fabrication of Superhydrophobic Graphene/Polystyrene Foams for Efficient and Continuous Separation of Immiscible and Emulsified Oil/Water Mixtures

Chunxia Zhao[1,3,*], Haoran Huang[1], Jiaxin Li[1], Yuntao Li[1,2,*], Dong Xiang[1,3], Yuanpeng Wu[1,2,3], Ge Wang[1] and Mingwang Qin[4]

[1]School of New Energy and Materials, Southwest Petroleum University, Chengdu 610500, China

[2]State Key Laboratory of Oil and Gas Reservoir Geology and Exploitation, Southwest Petroleum University, Chengdu 610500, China

[3]The Center of Functional Materials for Working Fluids of Oil and Gas Field, Sichuan Engineering Technology Research Center of Basalt Fiber Composites Development and Application, Southwest Petroleum University, Chengdu 610500, China

[4]School of Engineering, Southwest Petroleum University, Nanchong 637001, China

*Correspondence: polychem2011@hotmail.com (C. Zhao); yuntaoli@swpu.edu.cn (Y. Li)

Figure:

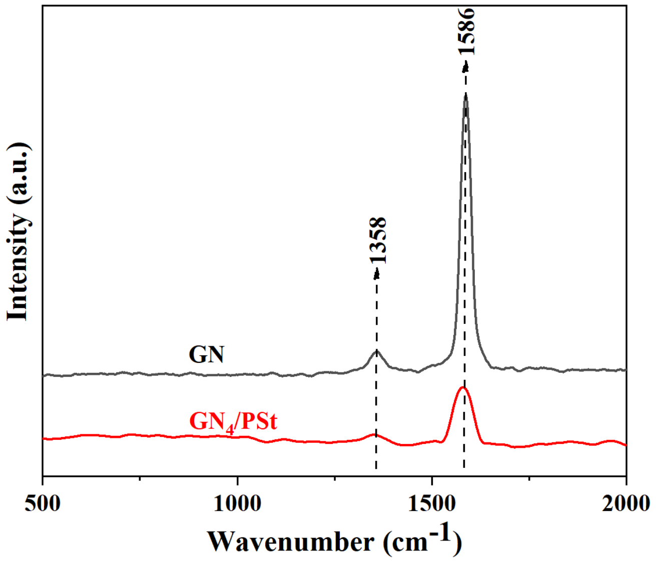

Figure S1. Raman spectra of GN and GN_4/PSt.

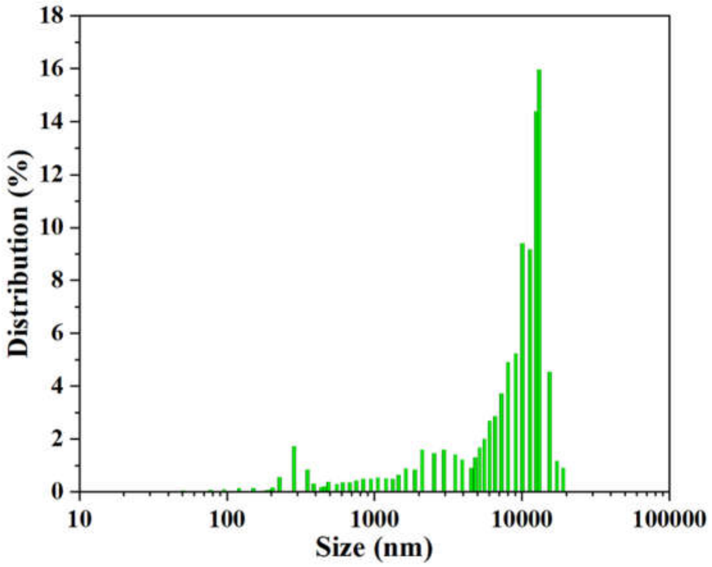

Figure S2. The pore size distribution of GN_4/PSt porous material.

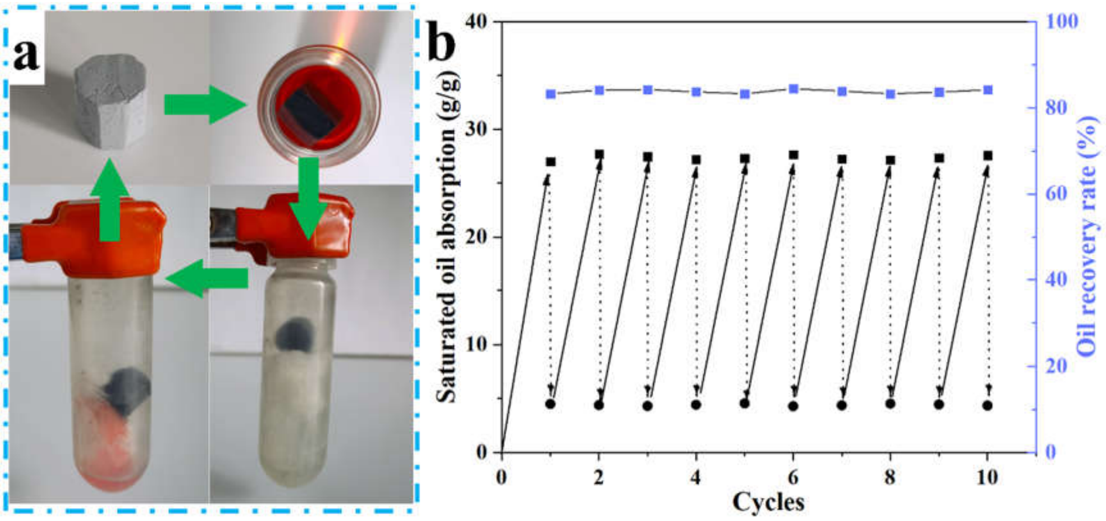

Figure S3. (**a**) The process of removal of petroleum ether from oil/water mixture and the regeneration of the foam. (**b**) Regeneration of GN_4/PSt sponge during the absorption of petroleum ether for 10 cycles.

Video:

Video 1: Continuous separation of immiscible light oil/water/heavy oil by GN_4/PSt porous material with the assistance of a pump.

Video 2: Continuous separation of surfactant-stabilized water-in-oil microemulsions with the assistance of a pump.
